# Enhanced Cycling Performance of the LiNiO_2_ Cathode in Li‐Ion Batteries Enabled by Nb‐Based Surface Coating

**DOI:** 10.1002/cssc.202402202

**Published:** 2024-12-10

**Authors:** Barbara Nascimento Nunes, Leonhard Karger, Ruizhuo Zhang, Aleksandr Kondrakov, Torsten Brezesinski

**Affiliations:** ^1^ Battery and Electrochemistry Laboratory (BELLA) Institute of Nanotechnology Karlsruhe Institute of Technology (KIT) Kaiserstr. 12 76131 Karlsruhe Germany; ^2^ BASF SE Carl-Bosch-Str. 38 67056 Ludwigshafen Germany

**Keywords:** Interfaces, Li-ion battery, Ni-rich cathode, Niobium, Protective coating

## Abstract

Lithium nickel oxide (LNO) is a promising cathode candidate in various next‐generation battery technologies. To increase its stability, doping and surface coating have become key strategies. Among various elements, niobium stands out for its dual role as an effective dopant and the advantages of its oxide phases as coatings. In this study, we explore Nb‐based coating of LNO, utilizing methods that minimize or eliminate solvent use. Additionally, the coated samples were treated at two different temperatures to study their effect on properties and electrochemical performance. Our results demonstrate that the coating process strongly affects the cell cyclability and further highlight the potential of Nb‐based protective coatings in enhancing LNO as a cathode active material for application in high‐energy‐density Li‐ion batteries.

## Graphical abstract

Nb‐based protective coating is explored to enhance LiNiO_2_ as a cathode active material for Li‐ion battery application. Effective coating involves thoroughly protecting the cathode particles, consuming surface impurities, and forming amorphous species of high ionic conductivity.

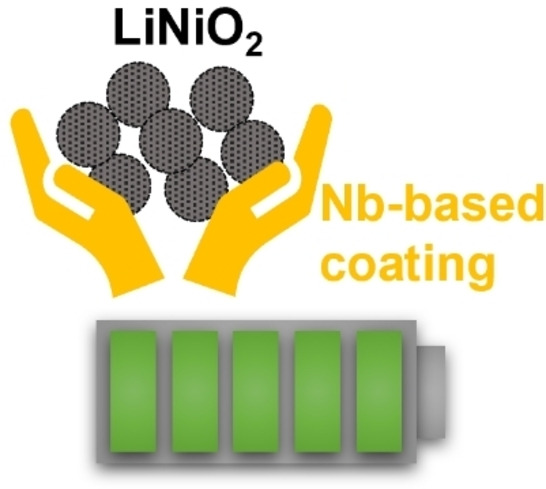

## Introduction

Li‐ion batteries (LIBs) have proven to be a promising choice for electric vehicles (EVs) due to their relatively high energy density, longevity, and reliability, having already demonstrated their value in portable devices.[^1^, ^2^, ^3^] Accordingly, key areas of advancements include improving the energy density and (charge) rate capability while reducing cost, particularly of the positive electrode (cathode), the most expensive component of LIBs.[^4^, ^5^] Cobalt, which is required in commercial cathodes like LiCoO_2_ (LCO), has become a critical material due to its high cost and associated ethical concerns.[^6^, ^7^] Thus, there has been a significant push to develop cobalt‐free cathodes, such as those based on LiNiO_2_ (LNO).

Since the 1990s, LNO has been recognized as a promising cathode active material (CAM), offering the advantages of a similar theoretical specific capacity (~274 mAh/g) and structure to LCO, but with higher Ni abundance.[^8^, ^9^, ^10^] Nevertheless, LNO faces significant challenges owing to structural and chemical instabilities, which significantly impair the overall electrochemical performance. Aside from its unstable lattice structure during (de)lithiation, LNO presents high reactivity with moisture and CO_2_, leading to structural changes and the formation of residual lithium species like Li_2_CO_3_, LiOH, or LiHCO_3_.^[11]^ The relatively high temperature used to its synthesis further intensifies this structural instability by causing cation mixing, where Ni^2+^ ions occupy positions in the lithium layer, resulting in incomplete lithiation and surface impurity formation.[^10^, ^12^, ^13^, ^14^] Other factors contributing to deteriorated performance include interfacial side reactions with the electrolyte, transition‐metal dissolution, oxygen release, and particle fracture.[^12^, ^13^] To address these challenges and achieve high stability, doping and surface coating are among the most prominent strategies employed. In general, doping enhances the structural integrity, while surface coating aims to build more stable interfaces and to reduce parasitic side reactions with the electrolyte.^[14]^

In a recent review, we discussed the use of niobium as a dopant and coating agent in layered transition metal oxides.^[15]^ Niobium has been highlighted in numerous studies for its benefits as an elemental dopant and for its various oxide phases as coating agents. In particular, several Nb‐based oxides (LiNbO_3_, Li_3_NbO_4_, Nb_2_O_5_, etc.) have been used as coatings to enhance cycling stability and rate capability by reducing surface/interface degradation.[^16^, ^17^, ^18^] LiNbO_3_ is the most prominent Nb‐based coating owing to its favorable properties and ease of synthesis using techniques such as sol‐gel or dry processing. Interestingly, in the context of LIB applications, while there are recent reports on niobium incorporation for LNO,[^19^, ^20^, ^21^, ^22^] specifically where Nb‐based modifications are made to the precursor CAM (pCAM), we could not find reports where coating methods were directly applied to LNO. For instance, Ober *et al*. presented a method for introducing niobium into nickel hydroxide [Ni(OH)_2_] pCAMs, resulting in the formation of intergranular Li_*x*_NbO_*y*_ phases as a kind of surface coating. Nevertheless, incorporating niobium and other high‐valence elements has been shown to decrease the primary particle size, and therefore, the better performance may not only correlate with the presence of a coating.[^21^, ^22^]

Here, different Nb‐based coating methods for LNO were tested, focusing on facile techniques that minimize or eliminate the use of solvents. Specifically, two methods were explored, namely dry coating (DRC) and incipient wetness impregnation (IWI). For DRC, LNO was ball milled together with hydrated niobium oxide (Nb_2_O_5_ ⋅ *n*H_2_O, niobic acid), chosen for its amorphous nature, which typically exhibits a higher specific surface area and reactivity than crystalline Nb_2_O_5_.[^23^, ^24^] IWI was selected, as it requires minimal solvent use to achieve uniform surface coverage through capillary action. Commonly used in catalysis to deposit metal nanoparticles, this method consists of filling the support‘s pore volume with an impregnating solution. The catalyst is then post‐treated (drying and annealing) to deposit the active material evenly onto the surface.[^25^, ^26^, ^27^] Furthermore, the coated samples underwent treatment at two different temperatures to explore their effect on properties and cycling performance.

## Results and Discussion

For the DRC method, hydrated niobium oxide was prepared by hydrolysis of NbCl_5_, and its amorphous nature was confirmed by X‐ray diffraction (XRD; see Figure S1, Supporting Information). Subsequently, the sample underwent ball milling (800 rpm, 10 min) to obtain more evenly sized and shaped particles, as observed by scanning electron microscopy (SEM; see Figure S2, Supporting Information). The fraction of niobium in Nb_2_O_5_ ⋅ *n*H_2_O was determined by inductively coupled plasma optical emission spectroscopy (ICP‐OES) to be 57.2 (±0.1) wt.%, suggesting a formula of Nb_2_O_5_ ⋅ 3H_2_O. The coated samples were prepared with 1.0 mol. % of niobium. Initially, two milling conditions were tested, namely 140 rpm for 30 min and 100 rpm for 10 min. SEM imaging indicated that reducing both rotational speed and milling time is effective in mitigating particle fracture (see Figure S3, Supporting Information). Therefore, milling conditions of 100 rpm for 10 min were selected to limit the energy input and prevent mechanical degradation (pre‐cycling) of the LNO secondary particles.

To ensure comparable conditions, the same fraction of niobium (1.0 mol. %) was used in the preparation of samples by the IWI method, from an ethanolic solution of Nb(OC_2_H_5_)_5_. A total of four samples were prepared, with subsequent heating at 400 and 700 °C under O_2_ atmosphere for each preparation method. Those produced by IWI are referred to as IMP400 and IMP700, while the ones prepared by DRC are labeled DRY400 and DRY700.

**Figure 1:**
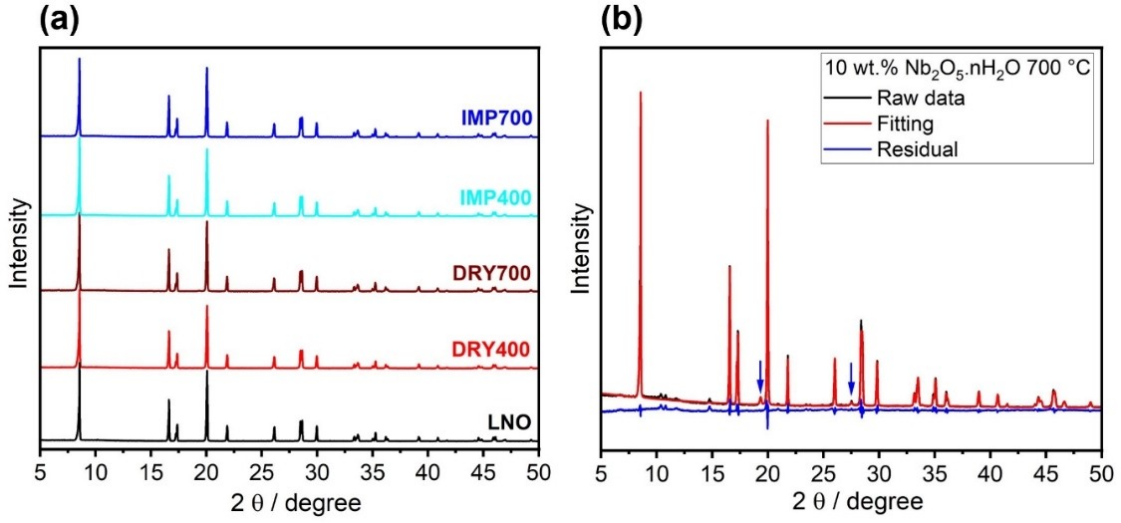
(a) Normalized XRD patterns of the bare and Nb‐coated LNOs and (b) Rietveld refinement profile for the LNO milled with 10 wt.% Nb_2_O_5_.*n*H_2_O and heated at 700 °C.

The bulk lattice of the bare and coated LNOs was probed using XRD, as shown in Figure 1a. The patterns confirm the presence of the *α*‐NaFeO_2_‐type structure within the *R−*3*m* space group for all samples. For the modified samples, no peak shifts or additional phases were observed, indicating that the coating does not alter the material‘s structure. For a more detailed investigation, samples with a higher coating content were prepared by milling the LNO particles with 10 wt.% of Nb_2_O_5_ ⋅ *n*H_2_O (6 mol. % Nb). Interestingly, Rietveld refinement analysis of XRD data collected from the material heated at 400 °C revealed a minor impact on crystal structure relative to that of the pristine LNO, as indicated in Table 1. Moreover, despite the high concentration of niobium oxide, no additional phases were observed for this sample (see Figure S4, Supporting Information). This may be due to the formation of amorphous Nb_2_O_5_ and potentially LiNbO_3_ at temperatures as low as 400 °C.[^28^, ^29^, ^30^, ^31^] The higher fraction of ${Ni}_{Li}^{•}$ defects compared to pristine LNO (2.1 % vs. 1.5 %) suggests that some lithium may be leached to the niobium oxide present on the surface to form LiNbO_3_. In contrast, when samples with 10 wt.% Nb_2_O_5_ ⋅ *n*H_2_O were heated at 700 °C, the cell parameters and point‐defect density increased significantly (see Table 1), especially the volume, indicating a possible incorporation of niobium into the LNO.^[32]^ Furthermore, reflections corresponding to other phases were observed (see Figure 1b); the more intense ones highlighted by arrows can be attributed to the formation of a rock‐salt material, fitting well with the disordered rock‐salt phase of Li_3_Ni_2_NbO_6_ (Li_2_Ni_1.333_Nb_0.667_O_4_, *Fm−*3*m* space group).^[33]^ This is reasonable given that Li_3_Ni_2_NbO_6_ is the product of the reaction between Li_3_NbO_4_, which typically forms from the conversion of LiNbO_3_ at temperatures around 700 °C,^[29]^ and NiO, which is commonly present on the surface of LNO.

**Table 1:** Structural refinement results for the bare LNO and 10 wt.% Nb_2_O_5_.*n*H_2_O‐coated LNOs heated at 400 and 700 °C.

Sample	${\mathrm{Ni}}_{\mathrm{Li}}^{•}$ /%	*V*/Å^3^	*a*/Å	*c*/Å	*R*_wp_/%
Bare LNO	1.5	101.4149	2.8732	14.1853	5.9941
Nb‐LNO, 400 °C	2.1	101.4759	2.8745	14.1813	6.8409
Nb‐LNO, 700 °C	7.0	101.9073	2.8793	14.1942	8.8376

**Figure 2:**
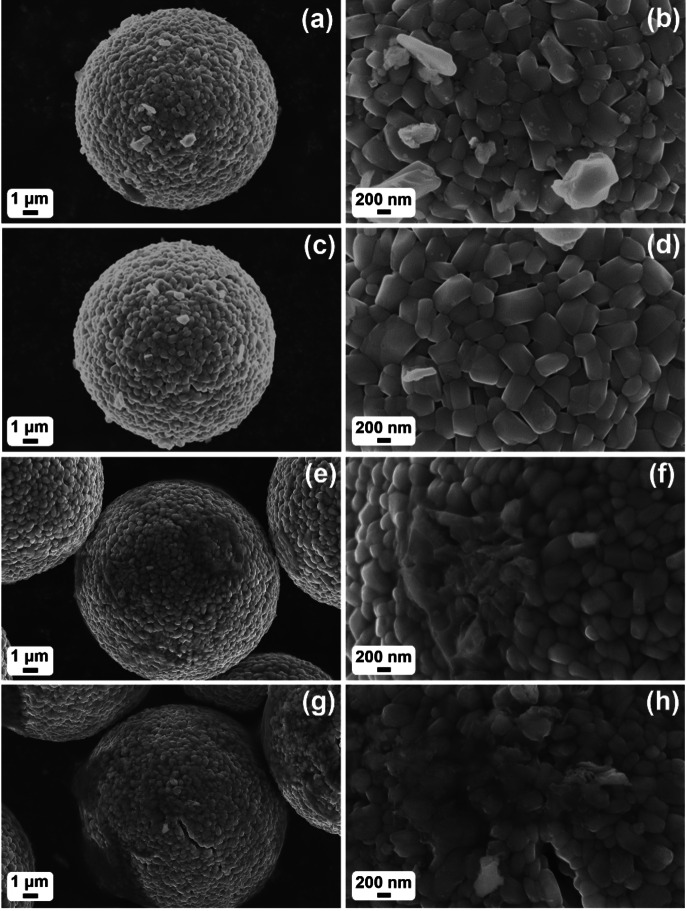
Low‐ and high‐magnification SEM images of (a, b) DRY400, (c, d) DRY700, (e, f) IMP400, and (g, h) IMP700.

Morphology and surface characteristics of the coated samples were examined by SEM. Overall, all of them are similar in terms of particle size, exhibiting spherical shapes typical of secondary particles.^[10]^ Higher‐magnification images clearly revealed that these secondary particles are composed of densely packed primary particles, which range in size up to several hundred nanometers. Notably, the two coating methods resulted in different appearances of the LNO surface. For the DRY400 (see Figures 2a and b) and DRY700 samples (see Figures 2c and d), small particles of Nb‐based material were visible on the top surface (as deduced from the contrast differences). Conversely, for the IMP400 (see Figures 2e and f) and IMP700 samples (see Figures 2g and h), the coating was not clearly distinguishable from the substrate, likely due to it being a thin film.^[34]^ However, in areas with material aggregation, the coating appeared more like a distinct layer on the LNO surface.

**Figure 3:**
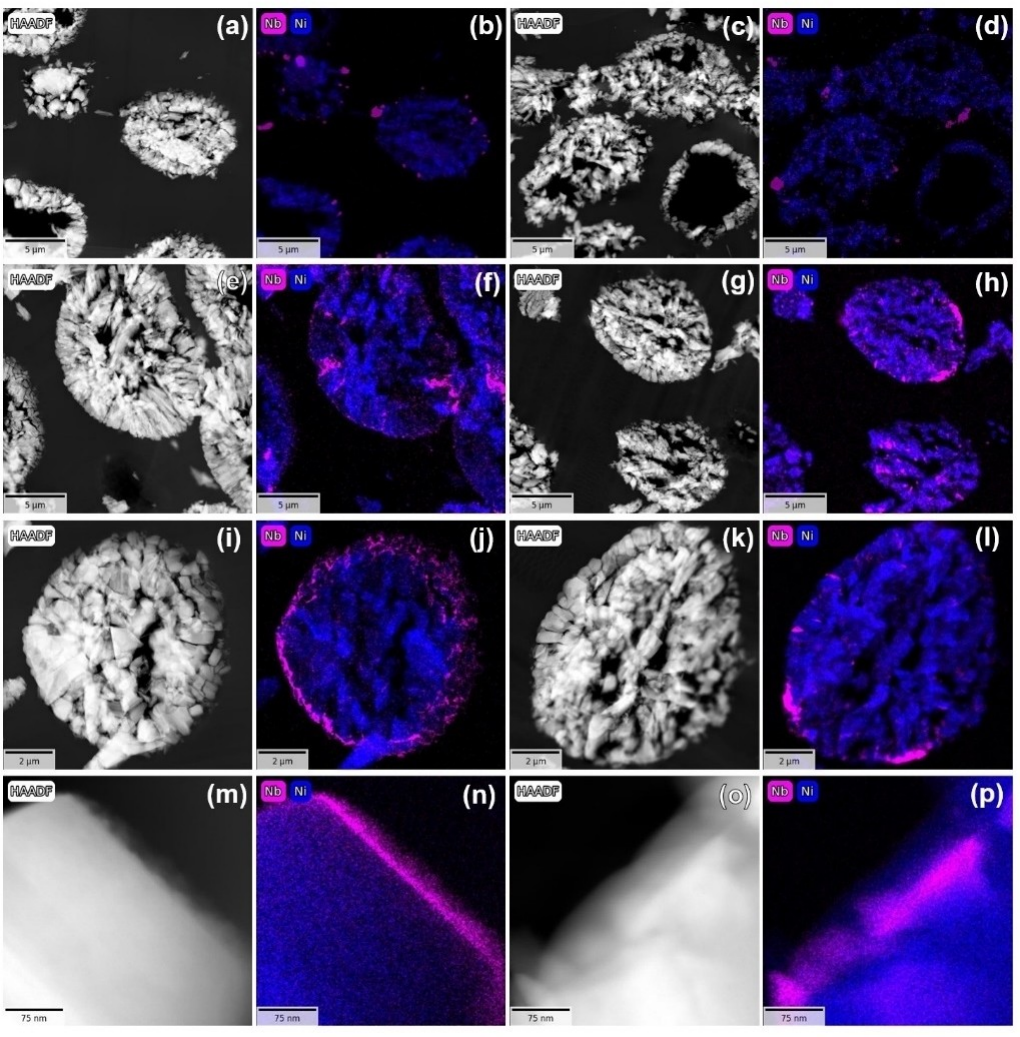
HAADF‐STEM images and corresponding elemental mapping of Nb and Ni for (a, b) DRY400, (c, d) DRY700, (e, f, i, j, m, n) IMP400, and (g, h, k, l, o, p) IMP700.

To verify the distribution of the Nb‐based coatings on LNO, elemental maps of niobium and nickel were acquired by high‐angle annular dark‐field scanning transmission electron microscopy (HAADF‐STEM) and energy‐dispersive X‐ray spectroscopy (EDS; see Figures 3a–p). The data revealed that, regardless of post‐treatment temperature, the DRC method results in niobium being detectable in specific areas (see Figures 3a–d) and exhibiting an agglomerate‐like shape, with only partial coverage of the LNO surface. Moreover, in agreement with the XRD results, selected area electron diffraction (SAED; see Figure S5, Supporting Information) confirmed that niobium is mostly present as an amorphous phase in DRY400, while DRY700 contains a crystalline, Nb‐based phase, possibly Li_3_NbO_4_. For the IMP400 and IMP700 samples, where the coating was applied using the IWI method, a more uniform surface coverage was observed, as evidenced in Figures 3e–l. Niobium was detected not only as a thin layer on the particle surface, but was also incorporated in the pore space. Furthermore, as apparent from the high‐magnification images in Figures 3m–p, unlike 400 °C, the higher temperature causes insertion of niobium into the subsurface regions.

**Figure 4:**
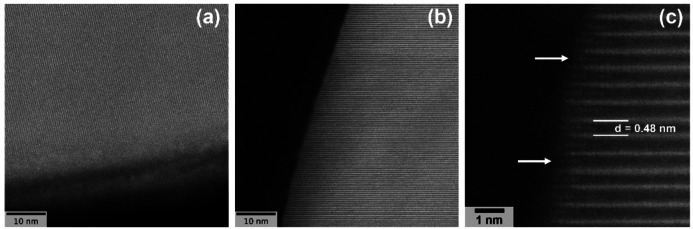
HRTEM images of (a) IMP400 and (b, c) IMP700. The TEM specimens were prepared by focused ion beam sectioning.

Due to the better niobium distribution across the LNO surface, the IMP400 and IMP700 samples were also examined by high‐resolution TEM (HRTEM; see Figures 4a–c). Both IMP400 (see Figure 4a) and IMP700 (see Figure 4b) exhibited the expected *c*‐planes of LNO (*R−*3*m* space group). For IMP400, amorphous material was found to be present on the surface. However, we note that this could also be the result of electron beam‐induced degradation. For IMP700, the *c*‐lattice parameter was close to that theoretically expected (0.47 nm), with a plane spacing of about 0.48 nm. Moreover, signs of niobium insertion into the subsurface were observed, as indicated in Figure 4c. The minor change in *d*‐spacing can be attributed to the fact that Nb^5+^ is heavier but smaller than Li^+^ and could be fit into the lattice without causing significant distortions (*r*_Nb5+_=0.64 Å vs. *r*_Li+_=0.76 Å).^[35]^ In addition, this kind of “doping” could lead to the formation of new phases, thus supporting the presence of a disordered rock‐salt phase (Li_3_Ni_2_NbO_6_) in the surface regions, as indicated by XRD.

**Figure 5:**
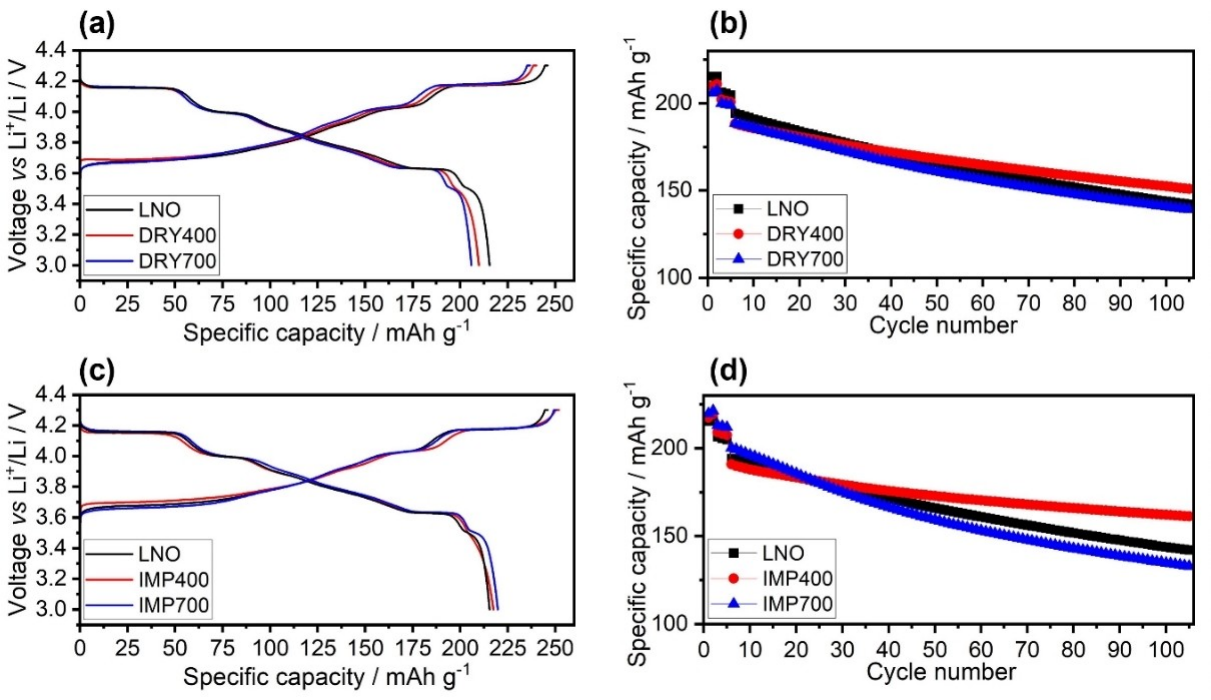
Electrochemical performance of LIB coin cells using the bare and Nb‐coated LNOs at 25 °C and in a potential window of 3.0–4.3 V vs. Li^+^/Li. (a, c) First‐cycle voltage profiles at 0.05 C and (b, d) specific discharge capacities against the cycle number, with the initial two cycles performed at 0.05 C, followed by three at 0.1 C and the subsequent ones at 0.5 C.

The electrochemical performance of the bare and coated LNOs (cathodes with 94 % CAM and 9 mg_CAM_/cm^2^ areal loading) was assessed by assembling coin cells with a lithium‐metal counter electrode. Figures 5a–d show the first‐cycle charge/discharge curves at 0.05 C rate (1 C=190 mA/g_CAM_) and the long‐term cycling performances at 0.5 C rate in a potential window of 3.0–4.3 V vs. Li^+^/Li. The presented data are representative of three individual cells for each material, which consistently demonstrated similar performance. In general, Figures 5a and c show similar voltage profiles for all samples, indicating that surface coating does not strongly affect the pristine LNO. However, the DRY400 and DRY700 cathodes exhibited higher overpotentials, particularly at the end of discharge, and delivered lower initial capacities compared to the bare LNO. Table 2 gives the initial specific charge and discharge capacities for the different samples, highlighting the impact of coating method and post‐treatment temperature. The DRY400 cathodes delivered a specific discharge capacity of 210 mAh/g_CAM_, while those using DRY700 achieved only 206 mAh/g_CAM_. In comparison, the bare LNO displayed a higher initial specific discharge capacity of 215 mAh/g_CAM_. In this case, the agglomerates present on the surface appear to lead to impedance buildup (hindered charge transfer), which is more pronounced at 700 °C, likely due to the formation of the mixed rock‐salt phase. By contrast, at the lower temperature of 400 °C, the formed phases are amorphous and do not strongly affect kinetics. For instance, amorphous LiNbO_3_ is known for its high ionic conductivity.[^15^, ^36^] For the IMP400 and IMP700 cathodes, the specific discharge capacities in the initial cycle were 218 and 220 mAh/g_CAM_, respectively. In these samples, the coating is thinner and more uniformly distributed across the LNO surface, thus not negatively affecting lithium transport. Notably, higher initial capacities were achieved than for the pristine LNO, particularly for IMP700. This is indicative of surface modification and is likely due in part to niobium incorporation, as observed by TEM, which apparently enhances the initial electrochemical performance (see first six cycles in which the C‐rate was increased from 0.05 C to 0.1 C, and finally to 0.5 C). However, the capacity retention after 100 cycles was inferior to that of LNO. In fact, as shown in Figures 5b and d, the post‐treatment temperature of 700 °C appeared to either have no or adverse effects on cycling performance, as the DRY700 and IMP700 cathodes showed capacity retentions of 74 and 66 %, respectively, compared to 73 % for the bare LNO. Interestingly, for DRY700, with the coating being more agglomerated and offering poor protection due to low surface coverage, the capacity retention was not affected much, whereas for IMP700, having the overall better coating, the retention was strikingly decreased. This result suggests that while a seemingly more uniform coating and a certain degree of niobium incorporation provide initially some beneficial effects such as improved kinetics, these advantages diminish over cycling, presumably due to partial degradation of the coating and increasing resistance at the electrode|electrolyte interface. From all samples tested in this work, the IMP400 cathodes exhibited the best capacity retention of 85 % after 100 cycles, consistent with the microscopy data indicating good surface coverage of the LNO secondary particles by an amorphous material. This means that, for IMP400, the coating is more effective in mitigating adverse side reactions with the electrolyte. Of note, amorphous, Nb‐based coatings are well documented in the literature for delivering satisfactory performance in Ni‐rich CAMs.^[15]^

**Table 2:** First‐cycle specific charge/discharge capacities and corresponding initial Coulomb efficiencies, as well as the 6^th^ and 106^th^ specific discharge capacities of the LIB coin cells discussed in Figure 5.

Sample	1^st^ *q*_ch_/mAh/g	1^st^ *q*_dis_/mAh/g	1^st^ *Φ*/%	6^th^ *q*_dis_/mAh/g	106^th^ *q*_dis_/mAh/g	Retention/%
Bare LNO	246.0	215.4	87.6	194.2	142.2	73.2
DRY400	240.1	209.8	87.4	188.4	150.9	80.1
DRY700	236.7	205.9	87.0	188.6	139.1	73.7
IMP400	252.0	217.5	88.3	190.9	161.3	84.5
IMP700	250.7	219.8	87.7	200.3	132.9	66.3

Interestingly, the DRY400 cathodes also showed superior cycling stability over the bare LNO, despite the relatively poor surface coverage. In this case, the amounts of Li_2_CO_3_ and LiOH surface impurities were also determined by acid titration (see Table S1, Supporting Information). Specifically, measurements were conducted on the pristine LNO and on samples produced by the DRC method before and after heating at 400 °C (DRY400). The results demonstrate that, even though the LNO particles were not properly coated, the DRC process (including post‐annealing) is capable of reducing the LiOH content in LNO by more than 50 %. This suggests that Nb‐based surface coating also helps lower the amount of residual lithium remaining from the synthesis.

**Figure 6:**
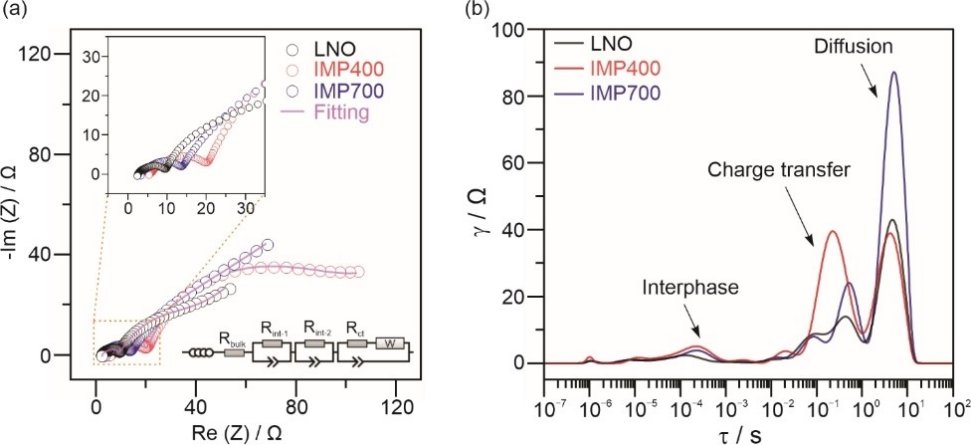
Kinetic analysis by EIS‐DRT for LNO, IMP400, and IMP700 after initial charging at 0.1 C rate with 1 h resting. (a) Nyquist plots of the electrochemical impedance and corresponding ECM fitting. Insets show a zoomed‐in view of the high‐frequency range and the model used in the fitting. (b) DRT patterns with peaks distinguished by characteristic time constants.

Given that IWI is the more effective coating method in this work, electrochemical impedance spectroscopy (EIS) measurements were conducted on the IMP400 and IMP700 cells to gain more insight into the electrochemical processes and cathode kinetics. Figure 6 shows Nyquist plots of the electrochemical impedance and corresponding distribution of relaxation times (DRT) patterns for the cells after the initial charge at 0.1 C rate. The open‐circuit voltages after equilibrium (1 h resting) maintained at the same value of about 4.2 V vs. Li^+^/Li. For quantitative analysis, equivalent circuit model (ECM) fitting was performed (see Figure 6a), with the extracted parameters given in Table S2 (Supporting Information). The DRT patterns, with characteristic peaks demonstrating the underlying kinetic processes in a model‐independent manner, are presented in Figure 6b.^[37]^ The major contributions are related to the interphase (with *τ*≈10^−5^–10^−2^ s), the charge transfer (with *τ*≈10^−2^–10^0^ s), and the bulk diffusion (with *τ*≈10^0^–10^2^ s). In agreement with the semicircles evident from the EIS data in Figure 6a and the cycling behavior in Figure 5c, the initial polarization (interphase contribution) was not strongly affected by surface coating. In contrast, the charge transfer between the electrode and the electrolyte manifested in a lower resistance for IMP700 compared to IMP400. As mentioned previously, the surface of IMP700 contains more lithium niobium oxide species (Li_3_Ni_2_NbO_6_, LiNbO_3_, etc.), which apparently promote ion transfer during the first charge cycle. Nevertheless, a higher degree of diffusive hindrance (inferior bulk diffusivity) was observed for IMP700, which may be due to differences in point‐defect density and/or the insertion of niobium into the CAM upon thermal treatment at 700 °C.

Overall, the results demonstrate that effective coating of LNO not only involves properly protecting the secondary particles, but also consuming surface impurities and forming amorphous coating species having a high ionic partial conductivity (e. g., LiNbO_3_) and favorable mechanical properties (without introducing more defects into the CAM).

## Conclusions

In this study, the secondary particles of LiNiO_2_ (LNO) were successfully modified with niobium at 1.0 mol. % by dry coating (DRC) and incipient wetness impregnation (IWI), followed by heating at different temperatures of 400 and 700 °C. XRD indicated that the bulk structure of samples annealed at 400 °C remains virtually unaltered. However, 700 °C leads to the appearance of side phases, including Li_3_Ni_2_NbO_6_. TEM studies further revealed that DRC produces samples, in which niobium is primarily detected in the form of agglomerates. By contrast, IWI facilitates more uniform coverage, with the higher annealing temperature causing niobium to insert, to some degree, into the subsurface regions of the LNO grains. Electrochemical testing revealed that the DRY400 and DRY700 cathodes exhibit higher overpotentials during cycling and deliver lower initial capacities than the bare LNO. For the IMP400 and IMP700 cathodes, higher capacities were achieved, particularly for the latter, suggesting that surface modification and partial niobium incorporation enhance the cyclability in the initial cycles. However, the 700 °C treatments were found to have either no or negative effects on longevity. IMP400 exhibited the overall best capacity retention after 100 cycles, indicating that the presence of a uniform, amorphous coating is most beneficial to the cycling stability. Interestingly, DRY400 also showed improved long‐term performance over the bare LNO cathode, despite the relatively low surface coverage. Acid titration measurements indicated that applying the DRC process with post‐annealing at 400 °C decreases the LiOH content in LNO by a factor of about two, with the coating acting as a kind of scavenger of residual lithium.

In summary, our study demonstrates that an effective Nb‐based coating for LNO involves thoroughly protecting the CAM particles, consuming surface impurities, and forming amorphous species of (ideally) high ionic conductivity.

## Experimental

*LNO preparation*: Ni(OH)_2_ pCAM was provided by BASF SE. LiOH ⋅ H_2_O was added to the pCAM considering a Li : Ni molar ratio of 1.01. The solids were mixed using a laboratory blender (Kinematica AG) for around 10 min, followed by transfer to a ceramic crucible. The heating conditions were 4 h at 400 °C and 6 h at 700 °C, with a heating rate of 3 °C/min, under O_2_ atmosphere (4 L/h). Sieving (32 μm) was performed inside an Ar glovebox.

*Dry‐coating (DRC) method*: First, amorphous niobium pentoxide (Nb_2_O_5_ ⋅ *n*H_2_O) was prepared by mixing 5.0 g of NbCl_5_ in 200 mL of deionized water and stirring rigorously for 3 h.^[24]^ The resulting solid was washed until a pH of 6 was reached, followed by drying at 70 °C. Then, Nb_2_O_5_ ⋅ *n*H_2_O was milled at 800 rpm for 10 min to obtain smaller and more uniform particles. LNO was blended with the proper amount of Nb_2_O_5_ ⋅ *n*H_2_O by ball milling at 100 rpm for 10 min. Subsequently, the coated CAM was heated at either 400 or 700 °C for 2 h under O_2_ atmosphere (7 L/h).

*Incipient wetness impregnation (IWI) method*: An ethanolic solution of 0.5 mol/L Nb(OC_2_H_5_)_5_ was gradually added to the LNO, in a ratio of 1 : 5 of solution to LNO (i. e., 0.4 mL for 2 g of LNO). The CAM was left to dry in an Ar glovebox, followed by heating at either 400 or 700 °C for 2 h under O_2_ atmosphere (7 L/h).

*Characterization*: Powder XRD patterns were obtained on samples placed in 0.03 mm glass capillaries (Hilgenberg) using a STADI P (STOE) diffractometer in Debye‐Scherrer geometry. Monochromatic Mo‐K_α1_ radiation (*λ*=0.7093 Å, 50 kV, 40 mA) and a Mythen 1 K detector (DECTRIS) were employed for the measurements. Rietveld refinement was carried out utilizing GSAS‐II,^[38]^ allowing variations in scale factor, zero shift, and size/strain broadening parameters. A fixed background was fitted to the data with a 12‐term Chebyshev polynomial function. In the structural model, the unit‐cell parameters, oxygen *z*‐coordinate, and isotropic atomic displacement parameters for each site were refined. SEM imaging was performed using a LEO‐1530 microscope from Carl Zeiss AG equipped with a field emission source. Acid titration measurements were performed using around 2 g of CAM mixed with 30 mL of deionized water. The suspension was filtered, and the remaining solid was rinsed with 20 mL of distilled water. The resultant solution was titrated with a 0.1 mol/L HCl solution using an Excellence Titrator (Mettler Toledo) equipped with a 20 mL burette (DV1020), a pH electrode (DGi111‐SC), and an automated OneClick™ system (method M828).

*Electrochemical testing*: For the electrochemical testing, a slurry was prepared by combining the CAM with Super C65 carbon additive and polyvinylidene difluoride binder (Solef 5130, Solvay) in a weight ratio of 94 : 3 : 3, respectively. The mixture was cast onto 0.03 mm‐thick aluminum foil using a stainless steel doctor‐blade of slit thickness 140 μm on an Erichsen Coatmaster 510 machine. Subsequently, the cathode tapes were dried under vacuum at 120 °C overnight, calendered at 14 N/mm (Sumet Messtechnik), and cut into circular 13 mm‐diameter electrodes. The areal loading was about 9 mg_CAM_/cm^2^. Coin cells were assembled using the prepared cathodes, a glass fiber GF‐D separator, LP57 electrolyte (1 M LiPF_6_ in a 3 : 7 ratio mixture of ethylene carbonate and ethyl methyl carbonate), and a lithium‐metal anode in an Ar glovebox. They were crimped at a pressure of 1 t. EIS measurements were performed after the first charge at 0.1 C rate with 1 h resting using a BioLogic SP‐300 potentiostat. The frequency range was set from 1 MHz to 100 mHz, with a 10 mV amplitude. An ECM model, as shown in Figure 6a, was used for curve fitting, including an inductive element (*L*), a resistor (*R*_Bulk_), three *ZARC* units (resistor *R* and constant phase element *Q* in parallel), and a Warburg element (*W*) to represent the interphase resistance (*R*_int_), the charge‐transfer resistance (*R*_ct_), and the diffusive contribution. Of note, the two semicircles appearing in the high‐frequency range (representing interphase contributions) cannot be easily distinguished, and therefore, are simply denoted as *R*_int‐1_ and *R*_int‐2_. DRT analysis was performed using a regularization parameter of 1.0 ⋅ 10^−5^.

## Conflict of Interests

The authors declare no conflict of interest.

1

## Supporting information

As a service to our authors and readers, this journal provides supporting information supplied by the authors. Such materials are peer reviewed and may be re‐organized for online delivery, but are not copy‐edited or typeset. Technical support issues arising from supporting information (other than missing files) should be addressed to the authors.

Supporting Information

## Data Availability

The data that support the findings of this study are available from the corresponding author upon reasonable request.
