# Supplementary material for: Enhanced Cycling Performance of the LiNiO2 Cathode in Li‐Ion Batteries Enabled by Nb‐Based Surface Coating
Source: ChemSusChem. 2024 Dec 10;18(8):e202402202. doi: 10.1002/cssc.202402202 (PMC11997913; doi:10.1002/cssc.202402202)
Supplement: Supplementary file 1 — Supporting Information [file CSSC-18-e202402202-s001.pdf]

ChemSusChem

Supporting Information

Enhanced Cycling Performance of the $LiNiO_2$ Cathode in Li-Ion Batteries Enabled by Nb-Based Surface Coating

Barbara Nascimento Nunes,* Leonhard Karger, Ruizhuo Zhang, Aleksandr Kondrakov,* and Torsten Brezesinski*

Supporting Information

Enhanced Cycling Performance of the $LiNiO_2$ Cathode in Li-ion Batteries Enabled by Nb-based Surface Coating

Barbara Nascimento Nunes,[a,*] Leonhard Karger,[a] Ruizhuo Zhang,[a] Aleksandr Kondrakov,[a,b,*] and Torsten Brezesinski[a,*]

[a] Battery and Electrochemistry Laboratory (BELLA), Institute of Nanotechnology, Karlsruhe Institute of Technology (KIT), Kaiserstr. 12, 76131 Karlsruhe, Germany.
[b] BASF SE, Carl-Bosch-Str. 38, 67056 Ludwigshafen, Germany.

*Email: barbara.nunes@kit.edu, aleksandr.kondrakov@basf.com, torsten.brezesinski@kit.edu

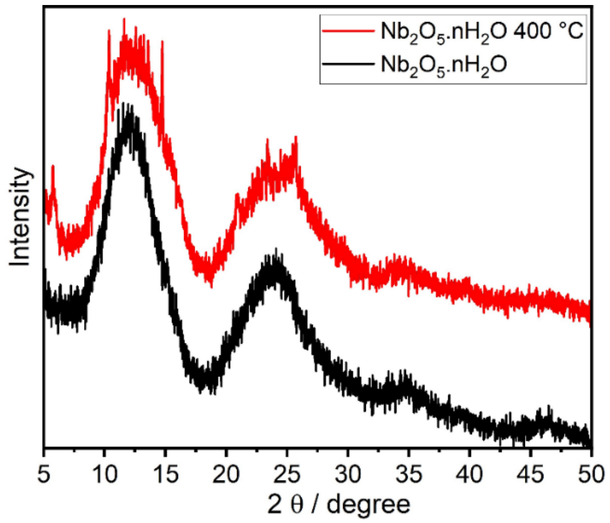

Figure S1. XRD patterns of $Nb_2O_5.nH_2O$ (a) before and (b) after heating under O_2 atmosphere.

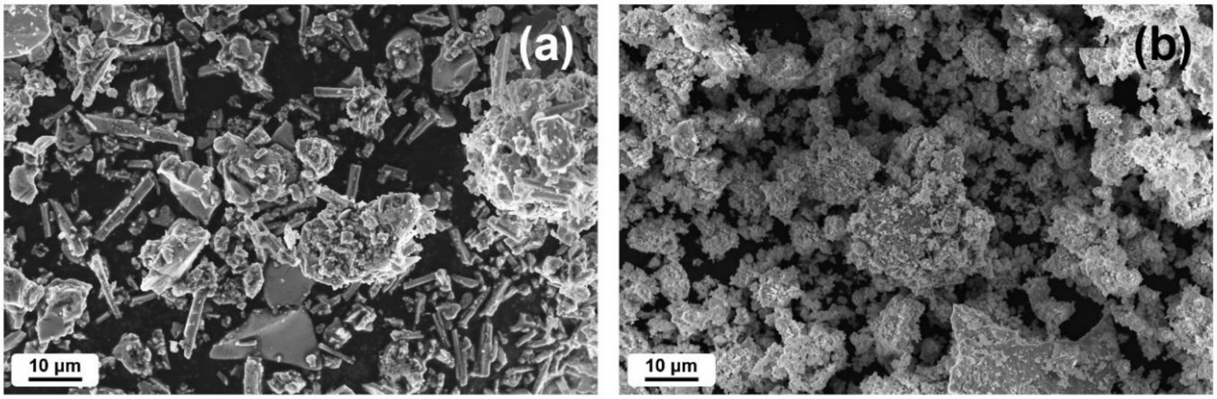

Figure S2. SEM images of $Nb_2O_5.nH_2O$ (a) before and (b) after ball milling at 800 rpm for 10 min.

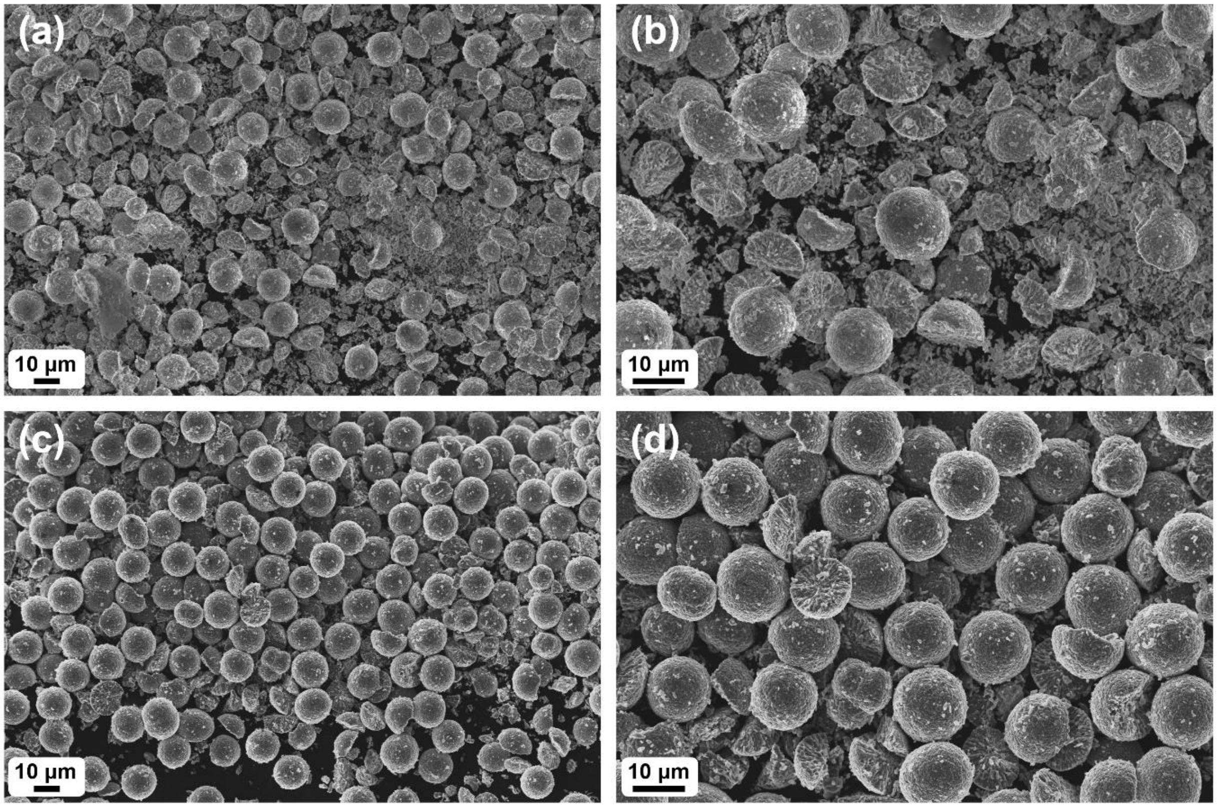

Figure S3. SEM images of DRY700 prepared using milling conditions of (a, b) 140 rpm for 30 min and (c, d) 100 rpm for 10 min.

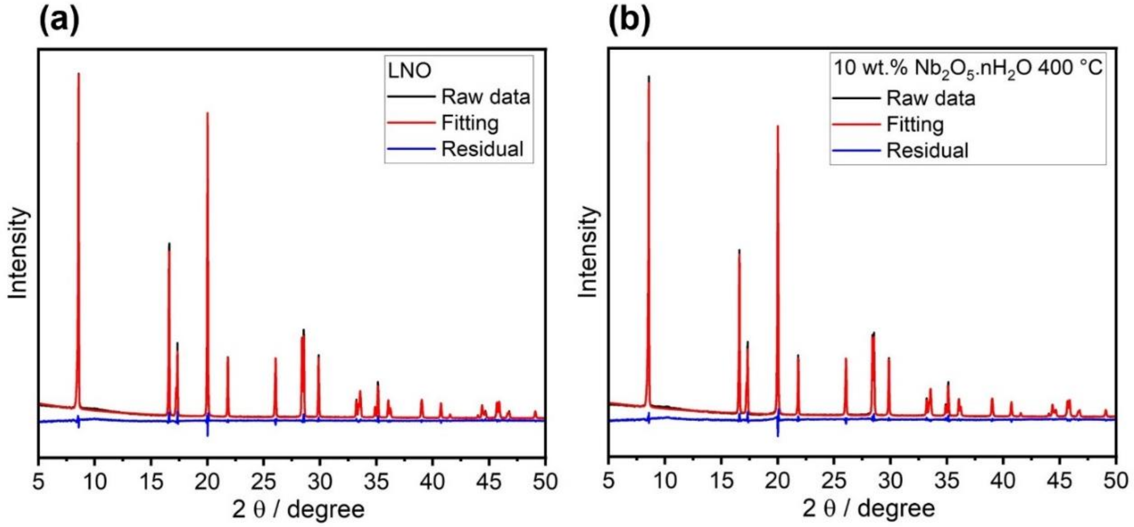

Figure S4. Rietveld refinements of XRD data collected from (a) bare and (b) coated LNO with 10 wt.% $Nb_2O_5.nH_2O$ heated at 400 °C.

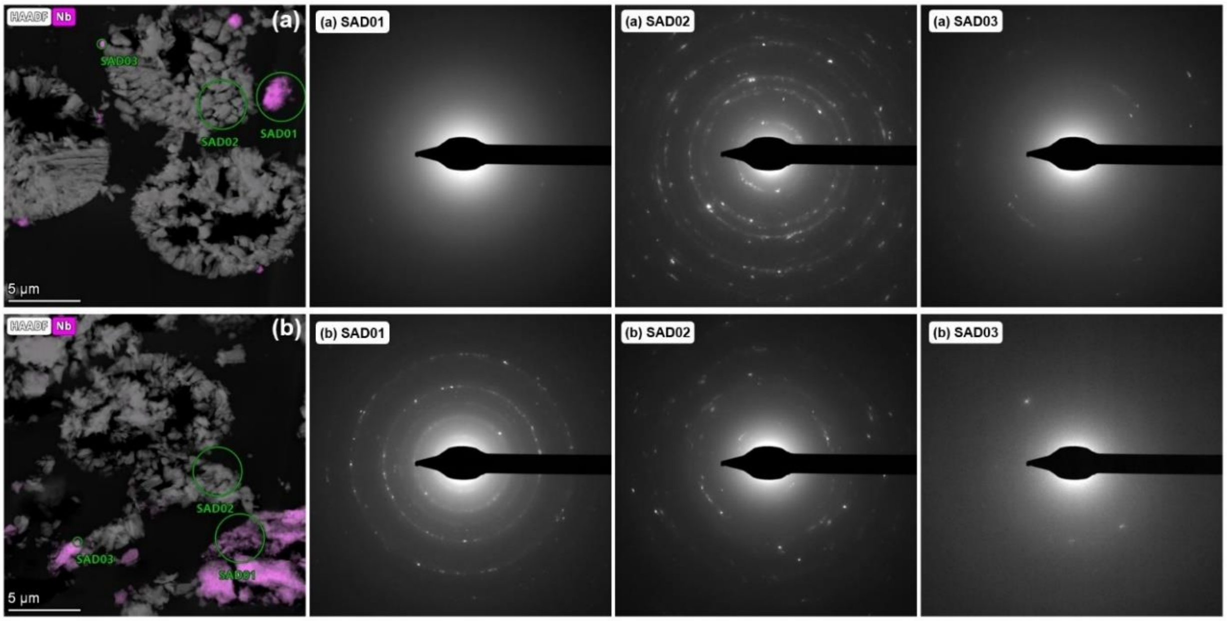

Figure S5. Elemental mapping and corresponding SAED patterns of (a) DRY400 and (b) DRY700. (a) SAD01, amorphous phase with ~3.8% Ni and ~96.2% Nb; SAD02, crystalline phase with ~99.9% Ni and ~0.1% Nb; and SAD03, amorphous phase with ~9.7% Ni and ~90.3% Nb. (b) SAD01, crystalline phase with ~8.3% Ni and ~91.7% Nb; SAD02, crystalline phase with ~99.5% Ni and ~0.5% Nb; and SAD03, crystalline phase with ~2.8% Ni and ~97.2% Nb.

Table S1. Results from acid titration measurements conducted on bare LNO and DRY400 before and after heating.

	**Li_2CO_3 (g/100 g)**	**LiOH (g/100 g)**
Bare LNO	0.429	0.854
LNO + 1 mol.% Nb	0.401	0.829
DRY400	0.434	0.372

Table S2. Extracted fitted parameters from EIS measurements.

	**Bare LNO**	**IMP400**	**IMP700**
L / H	$7.4 \cdot 10^{-8}$	$4.0 \cdot 10^{-8}$	$6.9 \cdot 10^{-8}$
R_{bulk} / Ω	2.3	5.4	3.1
R_{int-1} / Ω	3.7	6.5	4.4
CPE_{int-1} / F $s^{\alpha-1}$	$1.5 \cdot 10^{-4}$	$5.1 \cdot 10^{-4}$	$2.9 \cdot 10^{-4}$
α_{int-1}	0.69	0.57	0.63
τ_{int-1} / s	$1.9 \cdot 10^{-5}$	$4.5 \cdot 10^{-5}$	$2.8 \cdot 10^{-5}$
R_{int-2} / Ω	2.7	8.6	5.7
CPE_{int-2} / F $s^{\alpha-1}$	$4.8 \cdot 10^{-5}$	$9.9 \cdot 10^{-5}$	$9.2 \cdot 10^{-5}$
α_{int-2}	0.99	0.83	0.89
τ_{int-2} / s	$1.3 \cdot 10^{-4}$	$2.0 \cdot 10^{-4}$	$2.1 \cdot 10^{-4}$
R_{ct} / Ω	20.1	79.2	63.6
CPE_{ct} / F $s^{\alpha-1}$	$1.2 \cdot 10^{-2}$	$3.3 \cdot 10^{-3}$	$8.9 \cdot 10^{-3}$
α_{ct}	0.84	0.85	0.72
τ_{ct} / s	$1.8 \cdot 10^{-1}$	$2.1 \cdot 10^{-1}$	$4.5 \cdot 10^{-1}$
W / Ω $s^{-0.5}$	19.0	14.9	32.2
